# Strengthening Effect Evaluation of Developed Stiff-Type Polyurea Sprayed on Masonry Beam Surface Under Static Loading in Experimental and Numerical Tests

**DOI:** 10.3390/ma17215243

**Published:** 2024-10-28

**Authors:** Tae-Hee Lee, Jang-Ho Jay Kim

**Affiliations:** School of Civil and Environmental Engineering, Yonsei University, 50, Yonsei-ro, Seodaemun-gu, Seoul 03722, Republic of Korea; saintlth@yonsei.ac.kr

**Keywords:** strengthening effect, masonry structure, polyurea, stiff-type polyurea, static load, flexural load test, FEM simulation

## Abstract

Recently, deteriorated masonry structures aged over 30 years have shown serious structural problems. Simple and rapid maintenance plans are urgently needed for aging masonry structures. Polyurea (PU) is an effective retrofitting material for aging structures due to its easy spray application. This process saves time, reduces costs, and allows the structure to remain in use during retrofitting. However, a general PU is not suitable for retrofitting aged masonry and concrete structures due to its low stiffness. In this study, stiff-type polyurea (STPU) was selected as the reinforcement material for masonry structures. It was developed by modifying the chemical mix of general PU to improve stiffness. To evaluate the strengthening effect of STPU on masonry members under static loading, tests were conducted. The flexural load capacity of masonry beams with STPU-sprayed surfaces was assessed. Three different types of STPU applications were used to select the most efficient strengthening method. Reinforcing masonry structures with STPU allows brittle failure modes to achieve ductile behavior. This improves their structural performance under lateral stresses. The experimental data were used to calibrate FEM models for simulation. These models can be used for future parametric studies and masonry structural design.

## 1. Introduction

Recently, the number of deteriorated structures has been increasing, leading to a growing interest in methods for the repair and maintenance of deteriorated structures [1,2,3,4,5,6]. In Korea, masonry buildings constitute the largest proportion of deteriorated structures [7]. Masonry structures are widely utilized in construction projects around the world due to the easy availability of materials, their simplicity in construction, their long-lasting durability, and their fire resistance [8]. However, masonry structural members are known to have a very low tensile load-carrying capacity in tension since the brick–mortar interface layer is the weak link in the members [9,10,11]. In order to address the problems associated with aged masonry structures in South Korea, simple and rapid maintenance plans are urgently needed at present.

Fiber-reinforced polymers (FRPs) have been commonly used to reinforce masonry walls due to their high durability, exceptional strength, and lightweight properties [12,13,14,15]. Recently, among non-metallic fibers, CFRP (Carbon-FRP) has gained significant attention as a reinforcement material due to its high strength and corrosion resistance [16]. It has been increasingly used to reinforce structures in forms such as pre-stressed carbon-fiber laminations and CFRP grids [17,18]. However, the high cost of the material and concerns of over-reinforcement arise, as its strength may exceed that of masonry walls [19]. Also, FRP has the drawback of a potential loss of reinforcement effectiveness due to debonding, as well as the risk of the brittle failure of the masonry structure [20]. Polyurea (PU) has often been proposed as a reinforcement material to address the shortcomings of FRP. PU is a highly effective retrofitting material for aging structures due to its high-energy dissipation capacity, high ductility, and simplicity of application by surface spraying, which saves time and cost as well as usage of the structure while the retrofitting work is undergone [21,22,23,24,25]. There are concerns regarding polyurea coating. One concern is that although it can be easily applied in spray form, it requires a skilled professional. Another concern is that the material properties of polyurea vary significantly depending on the composition, and general PU is not suitable for retrofitting aged masonry and concrete structures due to its low stiffness [26,27].

Therefore, in this study, stiff-type polyurea (STPU), developed by modifying the chemical composition of general PU to enhance stiffness, was selected as the reinforcement material for masonry members [28]. STPU uses a prepolymer with higher strength than general PU and increases the NCO ratio to enhance the reaction with the hardener. As a result, the tensile strength increased, and elongation decreased, leading to a polyurea with improved stiffness [28]. The tensile strength, percent of elongation, and elastic modulus of STPU are 28 MPa, 250%, and 112 MPa, respectively, while those of general PU are 24 MPa, 310%, and 108 MPa [28]. To evaluate the strengthening effect of STPU for a masonry member under static loading, the STPU surface-sprayed masonry beams were tested to evaluate their flexural load-carrying capacity. Three different types of STPU applications were used to select the most efficient strengthening method. The final goal of this study was the validation of the proposed FEM models using ABAQUS/Standard 2022 software. Therefore, the experimental data were then used to calibrate FEM models for simulation, which can be used for future parametric studies and masonry structural design. Therefore, the following section will introduce the specimen details and the experimental set-up for the flexural load test, followed by an analysis of the test results. Additionally, the numerical modeling process will be explained, and the experimental results will be compared with the numerical model’s analysis.

## 2. Flexural Strength Test for STPU Surface-Sprayed Masonry Beam

### 2.1. Specimen Details

**Figure 1:**
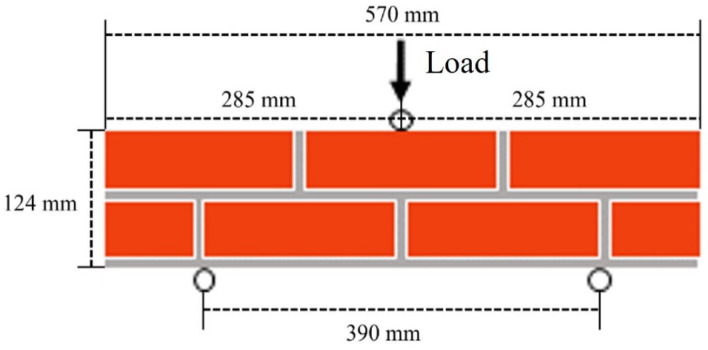
Masonry specimen details.

**Figure 2:**
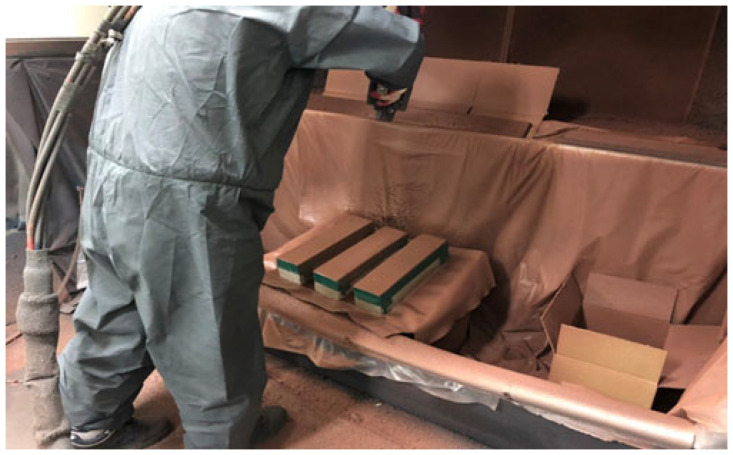
STPU application to specimen.

Red bricks with dimensions of 57 × 90 × 190 mm and a 10 mm mortar thickness were used for manufacturing the masonry member for the flexural strength test. To observe the reinforcing effect of STPU under extreme conditions, we simulated the critical situation where a masonry beam is subjected to a bending load. Figure 1 illustrates the effect of STPU reinforcement in various forms when applied to the masonry beam with low flexural strength in three-point bending tests. The masonry member was manufactured with a size of 124 × 90 × 570 mm and a span of 390 mm, and the 0.5B stacking method was used, as shown in Figure 1. Since the 0.5B stacking method is a method of stacking bricks in one row, the bricks used for the first floor were cut in half and used. The compressive strengths of the red brick and mortar used were 5.5 MPa and 22 MPa, respectively. STPU was applied with a thickness of 2 to 3 mm and at a distance of 60 cm from the specimen, as shown in Figure 2. STPU was continuously sprayed back and forth 8 to 10 times, and the application was carried out as uniformly as possible. Polyurea can be used under extreme conditions. However, in this study, the polyurea was applied by spraying, and both the spraying and testing were conducted at a temperature of 24 °C and a relative humidity of 65% [29].

**Figure 3:**
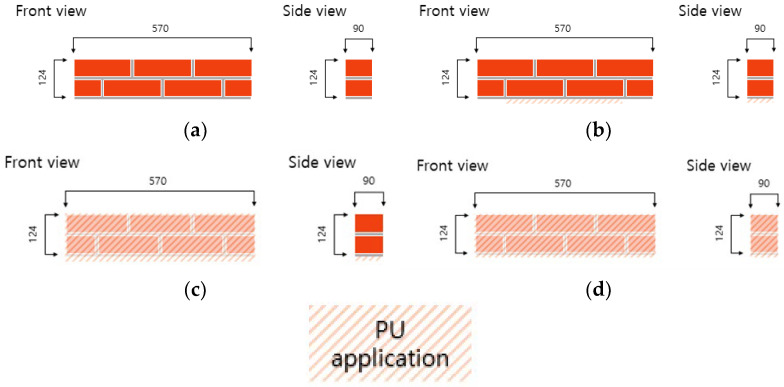
Specimen details for flexural strength test. (**a**) BFN. (**b**) BFP. (**c**) BFPS. (**d**) BFPF.

Figure 3 depicts the specimens utilized in the experiment, and different reinforcing methods were applied to each specimen. The specimens were labeled according to the reinforcing method used, as illustrated in Figure 3. “B” denotes the brick for the first character, and “F” denotes the flexure for the second character. “P” denotes the bottom surface strengthened by STPU, and “N” denotes the non-strengthened specimen for the third character. “S” denotes that the front and the back surfaces were strengthened by STPU, and “F” denotes that the front, the back, and both side surfaces were strengthened by STPU for the fourth character.

**Figure 4:**
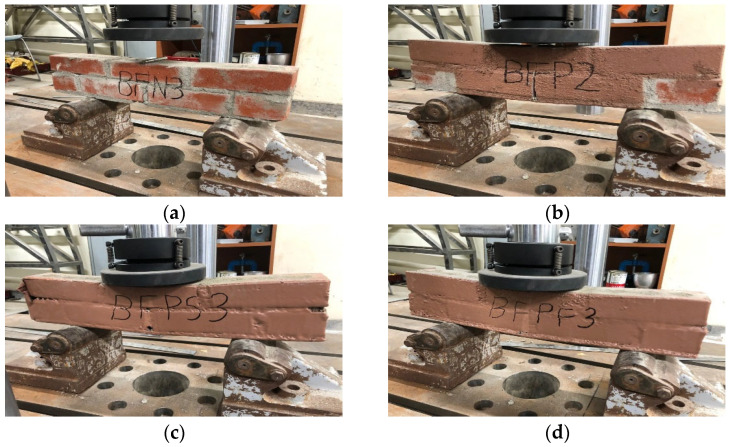
Flexural strength test set-up. (**a**) BFN. (**b**) BFP. (**c**) BFPS. (**d**) BFPF.

The flexural loading tests were performed according to the KSF 2408 standard [30] to understand the high flexibility effect of polyurea. The load device consisted of two support rollers and one material load roller to prevent the biases of the center in the vertical direction of the center point. The load was constantly applied at 1 mm/min for the displacement control method, and a test was conducted until the maximum displacement reached 8 mm. The equation for calculating the flexural strength when destruction occurs at the center point of the surface in the inter-tensile direction of the bending member is shown in Equation (1). Figure 4 is a photo of the flexural strength test set-up for each specimen.

(1) $$f_{b}=3Pl/2bh^{2}$$

Here, $f_{b}$: the flexural strength (MPa); P: the maximum load (N); l: the span (mm); b: the width of the fracture section (mm); and h: height of the fracture section (mm).

### 2.2. Flexural Strength Test Result

**Figure 5:**
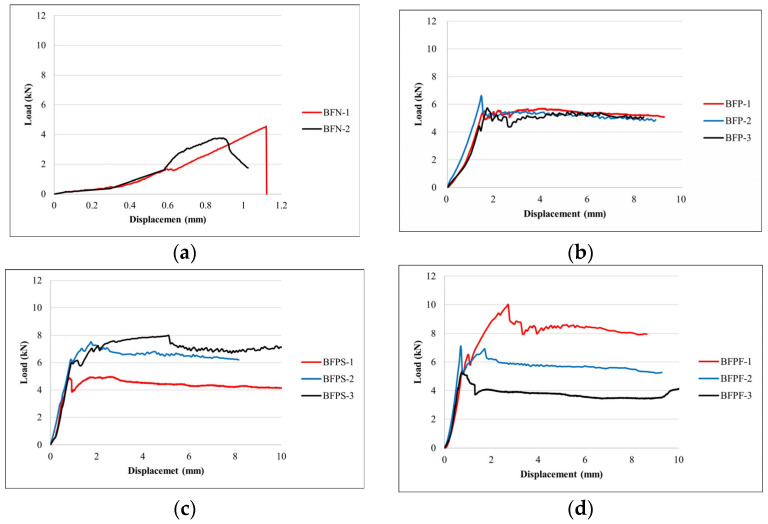
Load–displacement graph of the flexural strength test. (**a**) BFN. (**b**) BFP. (**c**) BFPS. (**d**) BFPF.

**Figure 6:**
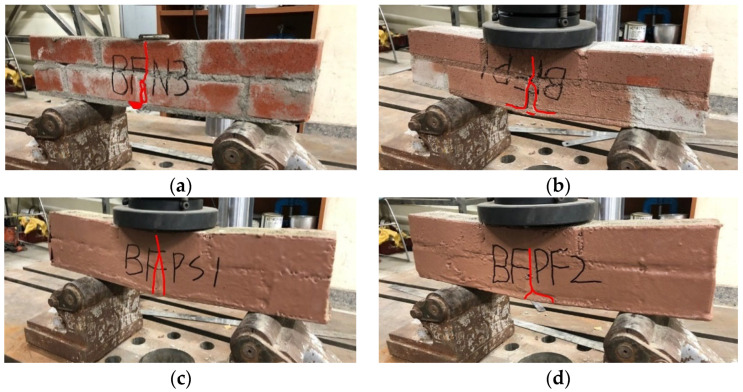
Crack pattern in flexural strength test specimens. (**a**) BFN. (**b**) BFP. (**c**) BFPS. (**d**) BFPF.

The maximum displacement in the BFN is the displacement at which the load can no longer be supported. In the BFP, BFPS, and BFPF specimens reinforced with STPU, the specimens still bore a load despite reaching the ultimate load. Determining the points at which the load could no longer be supported was difficult, so the maximum displacement was set as the point where the load started to decrease after peaking. The BFPF specimen exhibited the greatest improvement in ultimate load performance compared to the unreinforced BFN specimen, followed by the BFPS and BFP specimens. Compared to the BFN specimen, the BFP, BFPS, and BFPF specimens had 48%, 67%, and 103% higher ultimate loads, respectively. Table 1 and Figure 5 reveal that the unreinforced BFN specimen exhibited brittle failure, collapsing abruptly upon reaching the ultimate load with a maximum displacement of only 1 mm. Figure 5 demonstrates that the BFP, BFPS, and BFPF specimens, reinforced with STPU, exhibited ductile failure behavior (with an energy ratio over 75%) and could bear the load even after reaching the ultimate load capacity. The maximum displacement of these specimens ranged from 8.82 to 10.87 mm, which was 8.24–10.16 times that of the BFN specimen. Figure 6 depicts the crack patterns of the specimens after the flexural strength test.

The crack pattern of the unreinforced BFN specimen extends from the lower central mortar to the top central brick, with the lower mortar partially spalling. The BFP specimen, with its reinforced bottom parts, exhibited no spalling in the lower portions; instead, it had a crack pattern like that of the BFN specimen. The BFPS and BFPF specimens with reinforced front and back faces did not exhibit crack patterns, but a gap created in the lower part indicated that the internal masonry beam specimen initially failed, similar to the BFN and BFP specimens. Ductile behavior was then induced due to the high tensile strength of the STPU reinforcement. By reinforcing masonry structures with STPU, brittle failure modes can achieve ductile behavior, enhancing their structural performance under lateral stresses. Through this experiment, it is considered that reinforcing aging masonry structures with STPU can delay the collapse of masonry walls, thereby reducing the risk of casualties.

## 3. Numerical Evaluation for STPU Surface-Sprayed Masonry Beam

### 3.1. Numerical Modeling Method of Masonry Beam

**Figure 7:**
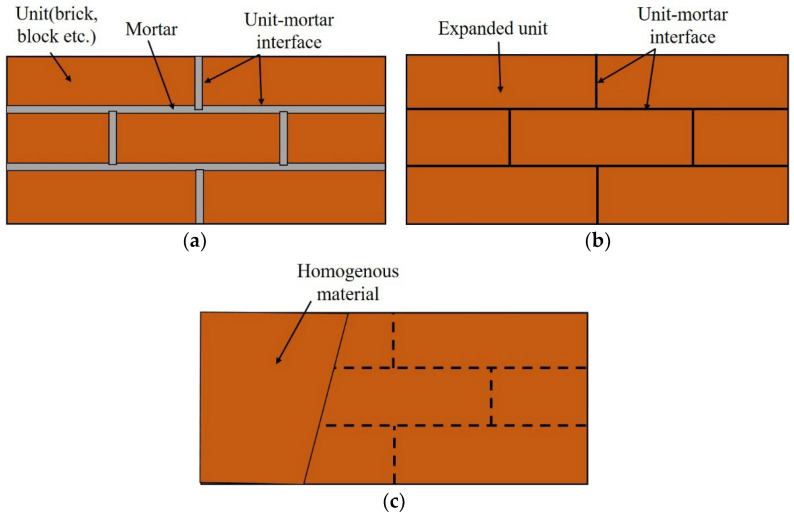
Finite element modeling methods of masonry wall. (**a**) Detailed micro-model. (**b**) Simplified micro-model. (**c**) Macro-model [31].

This section describes the numerical modeling and analysis of the flexural strength of masonry beams using ABAQUS software. A numerical analysis simulation is needed to determine the stability of STPU-reinforced masonry walls under different design variables. Two main methods are applied for numerically modeling masonry walls: the micro-method and the macro-method [31]. Figure 7 shows that the micro-method can also be broken down into a detailed micro-method and a simplified micro-method. The biggest difference between the micro- and macro-methods is whether the unit–mortar interface is involved. While the micro-method considers the unit–mortar interface and models each brick (unit) in a masonry wall, the macro-method models a masonry wall as a single homogenous material. The detailed micro-method models both the mortar and unit and can achieve the highest agreement with the actual masonry wall. However, the simplified micro-method models an expanded unit that considers the properties of the brick and the mortar and applies a unit–mortar interface to the unit-to-unit interface to reduce the analysis time. The micro-method is more accurate and allows the failure mode to be observed. However, it requires a longer analysis time. The macro-technique is commonly utilized to quickly analyze and verify the macroscopic structures. The micro-technique was utilized in this work to model the masonry wall and achieve great similarity to the actual experimental results. The flexural strength test was performed under static loading on a 0.12 m × 0.59 m × 0.09 m small structure; hence, the detailed micro-method was used.

### 3.2. Modeling and Variables of the Flexural Strength Test

**Figure 8:**
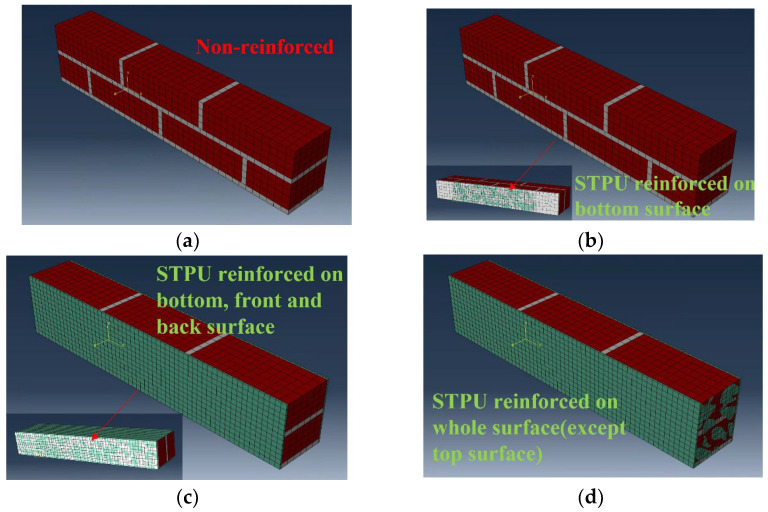
Finite element modeling of flexural strength test specimens. (**a**) BFN. (**b**) BFP. (**c**) BFPS. (**d**) BFPF.

**Figure 9:**
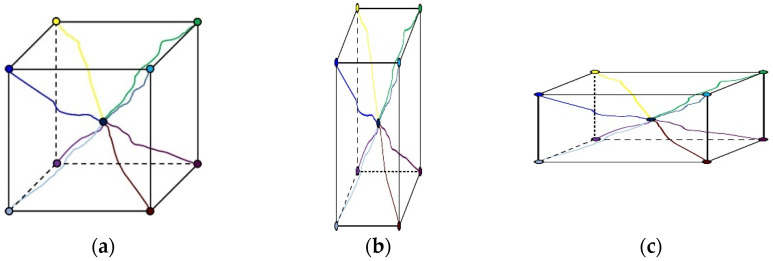
Eight chain rubber elasticity model. (**a**) Undeformed. (**b**) Tension. (**c**) Compression [36].

Flexural strength tests were modeled using the detailed micro-method, and the completed specimens are shown in Figure 8. The bricks and the mortar were made using a 3D model, whereas the STPU reinforcement on the bottom and sides of the specimens was made using a 2D shell model. The STPU used to reinforce the front and back of the specimens had a large surface area that touched the specimen, so it was modeled using a 3D model. The concrete damage plasticity (CDP) material model in Table 2 was used for the bricks and the mortar. Although originally designed for concrete, the CDP model’s parameters can be adjusted to approximate the behavior of other materials, such as brick and mortar, making it an appropriate choice for modeling masonry structures [32,33]. The CDP model was used to observe the failure modes and plastic deformation of the brick-and-mortar models. In order to simulate the STPU material, the Arruda–Boyce model was used as a constitutive model for STPU. Polymers, such as polyurea, have a chain-like molecular structure. It can attain a considerable strain rate of more than 100%. Moreover, it exhibits nonlinear hyper-elastic characteristics similar to those of rubber. Accordingly, a suitable numerical analysis model of polyurea is the Arruda–Boyce model, a highly accurate physical model formulated from a microstructure perspective [34,35]. The Arruda–Boyce model is based on a statistical-mechanical treatment of a material with a representative hexahedral volume element in which eight chains are diagonally connected, as shown in Figure 9. The material is assumed to be incompressible. The Arruda–Boyce model uses the strain energy potential as a function of strain to obtain its stress–strain relation, as shown in Equation (2) [36].

(2) $$U=\mu \left\{\frac{1}{2}\left(\bar{I_{1}}-3\right)+\frac{1}{20\lambda_{m}^{2}}\left({\bar{I_{1}}}^{2}-9\right)+\frac{11}{1050\lambda_{m}^{4}}\left({\bar{I_{1}}}^{3}-27\right)+\frac{19}{7000\lambda_{m}^{6}}\left({\bar{I_{1}}}^{4}-81\right)+\frac{519}{673750\lambda_{m}^{8}}\left({\bar{I_{1}}}^{5}-243\right)\right\}+\frac{1}{D}\left(\frac{J_{el}^{2}-1}{2}-\ln J_{el}\right)$$

Here, $\lambda_{m}$ is the elongation rate in longitudinal direction; $\bar{I}$ is the deviatoric strain invariant; $J_{el}$ is the elastic volumetric rate; and µ and D are the volume compression controlling material coefficients.

**Table 2:** Masonry wall material property for concrete damage plasticity [37,38].

	Density (kg/m^3^)	Elastic Modulus (MPa)	ν	ψ	ϵ	*σ_b_*_0_/*σ_c_*_0_	*K_c_*	μ
Brick	2100	5730	0.15	28	0.1	1.16	0.667	0
Mortar	2400	14,000	0.2	40	0.1	1.16	0.667	0

ν: Poisson’s ratio; ψ: dilation angle; ϵ: eccentricity; *σ_b_*_0_/*σ_c_*_0_: the compressive strength ratio in a two-axis state for the strength in a single-axis state; *K_c_*: parameter defining the shape of the surface of the plastic potential on a deviatoric plane; μ: viscosity parameter.

**Figure 10:**
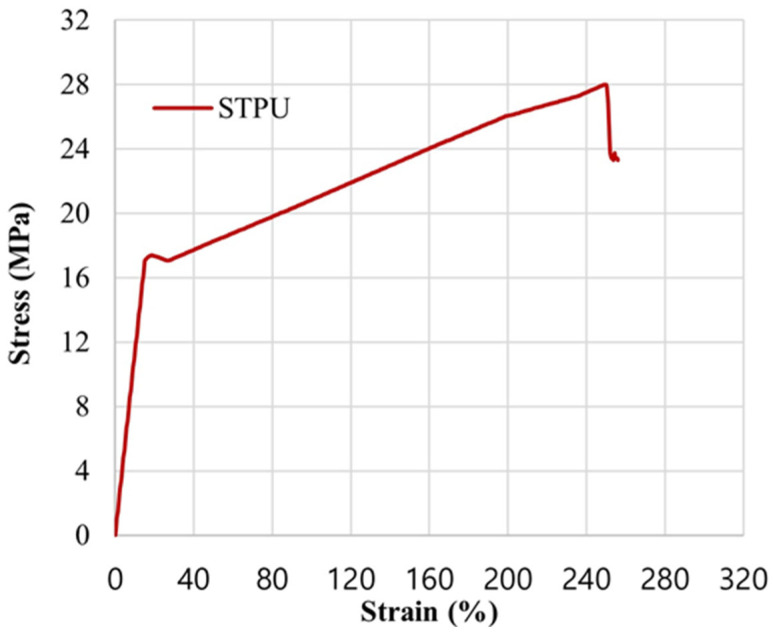
Strain–stress graph of STPU [28].

To reflect the physical properties of STPU in the numerical analysis, the strain–stress data obtained from the tensile strength test were applied to the material model, as shown in Figure 10 [28]. The properties of joint interfaces between the beam and STPU used in the simulation are summarized in Table 3.

**Figure 11:**
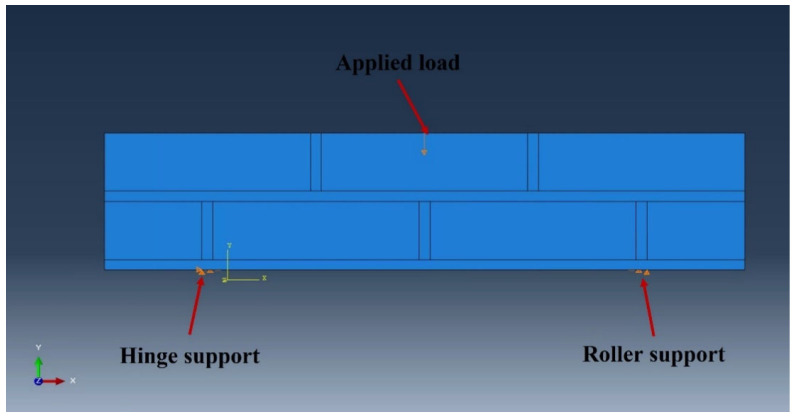
Boundary condition of masonry beam modeling.

The flexural strength tests were performed under three-point bending, and the boundary conditions described in Figure 11 were applied. As shown in Figure 11, the loading was controlled by displacement. To apply the load, a 13 mm displacement was applied under the boundary condition. Explicit analysis was utilized because the results were not linear, and our interest was in observing severe damage (element separation) beyond plastic deformation. The general contact (explicit) in ABAQUS was used to create the interface between the mortar and the brick and an unstructured mesh was used to model the nonlinear behavior of the mortar. The mesh size of the brick and mortar was 50 mm, with eight-node element types of C3D8R applied. The 30 mm mesh size and the C3D8RH element type were applied for STPU.

### 3.3. Numerical Analysis of Flexural Strength Test

**Figure 12:**
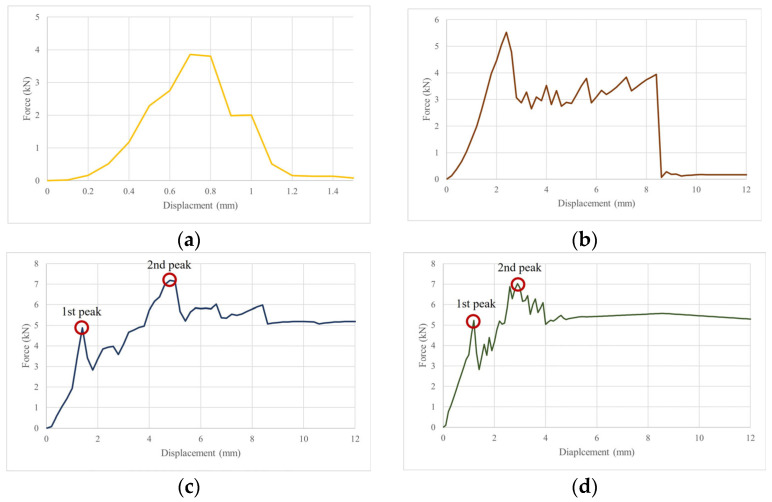
Force–displacement graph of numerical analysis. (**a**) BFN. (**b**) BFP. (**c**) BFPS. (**d**) BFPF.

**Figure 13:**
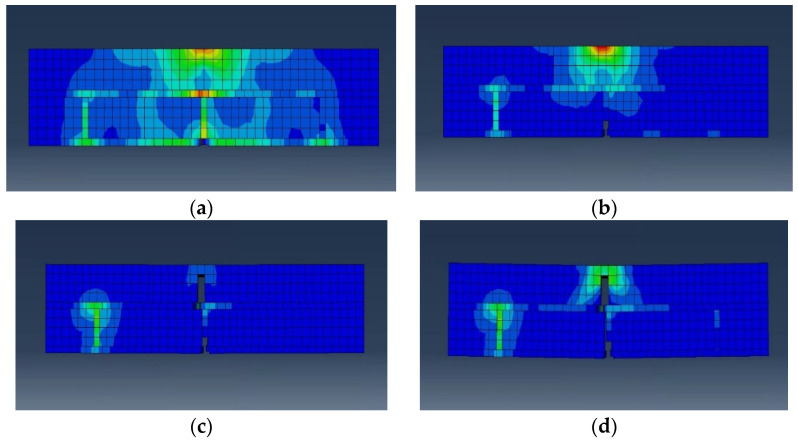
Element spalling progress in BFN specimen. (**a**) First element spalling. (**b**) Element spalling progress-1. (**c**) Element spalling progress-2. (**d**) Element spalling progress-3.

**Figure 14:**
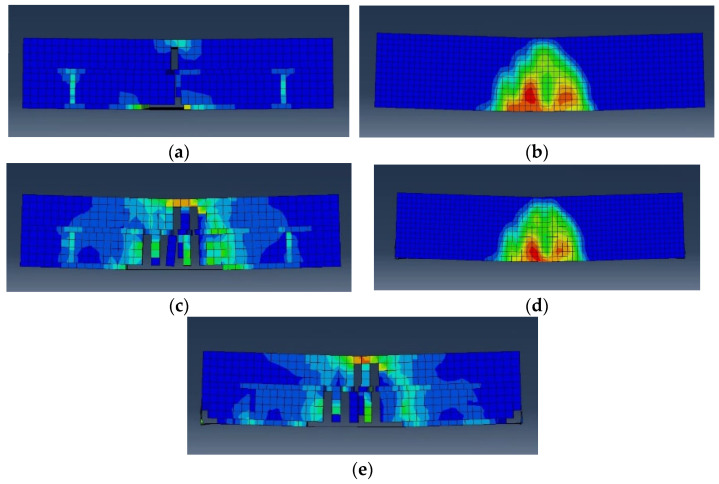
Element spalling progress in BFP, BFPS, and BFPF specimens. (**a**) BFP. (**b**) BFPS. (**c**) Inside of BFPS. (**d**) BFPF. (**e**) Inside of BFPF.

Figure 12 shows a summary of the results of the numerical analysis of the flexural strength test performed using ABAQUS. Figure 13 and Figure 14 show the element failure and damaged shapes after the load was applied. Figure 12 shows that the maximum load occurred in the following order: BFN followed by BFP, BFPS, and BFPF. The maximum load difference between BFPS and BFPF was small, measuring only 0.16 kN. The reinforcing effect of STPU was proven, with the maximum displacement that could withstand the load being 0.8 mm in BFN and 8.4 mm in BFP. Moreover, the maximum load in BFP was higher than that in BFN. As shown in Figure 12b, the BFP specimen, reinforced with STPU only in the lower part, could withstand a load of up to 8.4 mm after the maximum load. Presumably, the lower part of the masonry wall fails at the maximum load, which can explain this phenomenon. After withstanding a 3–4 kN load under the reinforcing effect of STPU, cracks that started in the lower part of the wall spread to the upper part, which could no longer hold additional loads. As shown in Figure 12c,d, when the front and back of the specimen were reinforced with STPU, the first and second peaks were observed in the force–displacement graph, which is presumably due to failure in the lower part of the masonry wall at the first peak. Moreover, the load was supported by the tensile strength of STPU until cracks gradually progressed to the upper part, leading to failure in the upper part at the second peak. The numerical analysis graph of BFN in Figure 12a is similar to the BFN-2 graph in Figure 5a; the graph in Figure 12b exhibits a similar trend as the BFP-2 graph in Figure 5b; the graphs in Figure 12b,c exhibit similar behaviors as the BFPS-3 and BFPF-1 graphs in Figure 5c,d, where the first and second peaks were observed, respectively.

Figure 13a shows that the first element failure occurred in the central lower part of the mortar in the BFN specimen. The element failures in Figure 13b–d reveal that the failures progressed toward the central upper part as displacement increased. Figure 13 shows that the pattern of cracks observed via numerical analysis is similar to that in Figure 6a, which presents the actual experimental results. Figure 14a shows that the BFP crack pattern, which shows a similar crack pattern to that of BFN but with more elements in the lower part, failed. BFPS and BFPF reinforced with STPU on the front and back exhibited failure patterns that progressed not only in the central part but also to the sides in the internal section of the masonry wall, as shown in Figure 14c,e.

Table 4 lists a summary of the mean values of the data from the experiment and the numerical analysis, as well as the ratio of the numerical analysis results to the experimental data. Analysis of the ultimate load revealed that BFPF was off by 16%, whereas the other specimens were off by less than 10%. For BFN and BFP, the maximum displacement error was 25%, and for BFP, it was 5%. For BFPS and BFPF, the load continued to be supported up to 13 mm of displacement, which prevented observations of the maximum displacement. Because the BFPF-1 specimen had a relatively higher ultimate load than the other specimens in the real experiment, the difference between the experimental data from the three specimens and the load value from the numerical analysis could be lessened by averaging more experimental data. Because the load was carried even after maximum displacement in the real experiment, the numerical analysis was deemed to accurately reflect the ductility of the STPU-reinforced specimens. Due to the nonlinear nature of the materials in the masonry wall, errors can occur. However, the reinforcing effect of STPU and the load–displacement behavior of each specimen observed in the experiment were also observed in the numerical analysis, indicating that the numerical analysis modeling of the flexural strength test developed in this study could be used for the masonry wall.

## 4. Conclusions

To evaluate the strengthening effect of STPU on the masonry wall, a flexural strength test was conducted. From the flexural strength test results, the strengthening effect of STPU was higher in the order of BFPF, BFPS, BFP, and BFN. The STPU-strengthened specimen showed ductile behavior after the ultimate load, whereas the non-strengthened specimen showed brittle fracture.

As the reinforced surface increased, the ultimate flexural load also tended to rise. It is considered that as the surface area reinforced with PU increased, the adhesive performance of the PU improved, leading to load dissipation rather than concentration. It was found that the reinforcement effect was superior when the entire surface of the beam was reinforced rather than only the bottom surface, which is the most critical section. When reinforced with STPU, the flexural load increased by approximately two times compared to the unreinforced specimens, but the reinforcement effect is still significantly lower than that of steel or FRP [41].

For the flexural strength test, to construct a numerical model of the STPU-strengthened masonry wall using ABAQUS, the Arruda–Boyce model was applied to STPU, and a detailed micro-model was applied to the masonry wall. The difference ratio between the simulation and the experimental result was under 16%. The simulation results showed the same crack patterns and tendency as the experimental result. Considering the nonlinear material properties of the masonry beam, it can be concluded that the numerical model of the masonry beam reinforced with STPU was successfully established. Since masonry structures are residential facilities, fire-resistance testing should be included in future studies.

## Figures and Tables

**Table 1:** Flexural strength test results.

	Test Specimen Number	Ultimate Load (kN)	Maximum Displacement (mm)	Flexural Strength (MPa)
BFN	1	-	-	-
	2	3.75	1.02	1.59
	3	4.54	1.12	1.92
	Average	4.15	1.07	1.75
BFP	1	5.70	9.23	2.41
	2	6.94	8.87	2.93
	3	5.74	8.36	2.43
	Average	6.13	8.82	2.59
BFPS	1	4.88	12.70	2.06
	2	7.61	8.16	3.22
	3	8.24	11.74	3.48
	Average	6.91	10.87	2.92
BFPF	1	10.03	8.68	4.24
	2	6.93	9.29	2.93
	3	8.24	10.26	3.48
	Average	8.40	9.41	3.55

**Table 3:** Properties for joint interfaces [39,40].

	Tangential Behavior	Cohesive Behavior		
	Friction Coefficient	Stiffness of Joint in the Normal Direction *K_nn_* (N/mm^2^)	Stiffness of Joint in the First Shear Direction K_ss_ (N/mm^2^)	Stiffness of Joint in the Second Shear Direction K_tt_ (N/mm^2^)
Brick and Mortar	0.75	35	14.42	41.42
Brick and STPU	0.06	41.6	17.5	17.5

**Table 4:** Results comparison between experiment and numerical analyses.

Specimen	Data Type	Ultimate Load (kN)	Maximum Displacement (mm)	Flexural Strength (MPa)
BFN	Numerical	3.86	0.80	1.63
	Experimental	4.15	1.07	1.75
	Ratio	0.93	0.75	0.93
BFP	Numerical	5.52	8.40	2.34
	Experimental	6.13	8.82	2.59
	Ratio	0.90	0.95	0.90
BFPS	Numerical	7.18	-	3.04
	Experimental	6.91	10.87	2.92
	Ratio	1.04	-	1.04
BFPF	Numerical	7.03	-	2.97
	Experimental	8.40	9.41	3.55
	Ratio	0.84	-	0.84

## Data Availability

The original contributions presented in the study are included in the article, further inquiries can be directed to the first author.
